# A scheme for enabling the ultimate speed of threshold switching in phase change memory devices

**DOI:** 10.1038/s41598-021-85690-9

**Published:** 2021-03-17

**Authors:** Nishant Saxena, Rajamani Raghunathan, Anbarasu Manivannan

**Affiliations:** 1 Phase Change Memory Lab, Advanced Memory and Computing Group, Department of Electrical Engineering, Indian Institute of Technology Madras, Chennai, 600036 India; 2 UGC-DAE Consortium for Scientific Research, DAVV Campus, Khandwa Road, Indore, Madhya Pradesh 452001 India

**Keywords:** Electronic devices, Information storage

## Abstract

Phase change materials exhibit threshold switching (TS) that establishes electrical conduction through amorphous material followed by Joule heating leading to its crystallization (*set*). However, achieving picosecond TS is one of the key challenges for realizing non-volatile memory operations closer to the speed of computing. Here, we present a trajectory map for enabling picosecond TS on the basis of exhaustive experimental results of voltage-dependent transient characteristics of Ge_2_Sb_2_Te_5_ phase-change memory (PCM) devices. We demonstrate strikingly faster switching, revealing an extraordinarily low delay time of less than 50 ps for an over-voltage equal to twice the threshold voltage. Moreover, a constant device current during the delay time validates the electronic nature of TS. This trajectory map will be useful for designing PCM device with SRAM-like speed.

## Introduction

In view of an exponential growth of global data storage and the physical limit of conventional transistor scaling in recent years, tremendous efforts have been devoted primarily to the fundamental re-thinking of enabling high-performance storage class memory, and also on the unconventional information processing capabilities^1–4^. Chalcogenide-based phase change memory (PCM) technology has been successfully demonstrated as a promising candidate for next-generation high-speed, non-volatile memory devices. It utilizes the contrast between a high-resistance amorphous (*reset* state, binary ‘0’) and a low-resistance crystalline (*set* state, binary ‘1’) state of phase change material^5^. In PCM programming, the *set* state is achieved by the application of nanosecond (ns)/picosecond (ps) voltage pulses, however, it is essentially a time limiting process, involving two steps. At first, the threshold switching (TS) takes place above a critical voltage known as the threshold voltage (*V*_*T*_), characteristic of the active material, during which rapid breakdown of electrical resistance from amorphous-off (a-off) to a conducting (a-on) state is evidenced by a steep rise in the device current (*I*_*d*_)^6^. This enables effective Joule heating leading to crystallization of the active material forming the *set* state. On the other hand, the *reset* operation can be achieved in a single step by a rapid melt-quench process usually by applying a *reset* pulse of much shorter pulse-width compared to the *set* pulse as demonstrated within as fast as 400 ps^7^. Thus, the operating speed of PCM is mainly controlled by the speed of *set* operation. Recently, the emphasis has been given on enabling faster *set* operation in sub-ns timescales; by controlling the nucleation process through alloying strategy i.e. Sc_0.2_Sb_2_Te_3_ and also incubation voltage assisted pre-structural ordering^8,9^. Moreover, ultrafast crystallization of phase change materials has been demonstrated in few ps by means of femtosecond X-ray diffraction or electromagnetic waves^3,10^. Therefore, even though phase change materials have the ability to crystallize at extremely fast timescales, the realization of a fast *set* process in case of electronic memories is hindered by TS. This is because of the fact that Joules heating becomes effective only soon after the TS due to rapid current flow in the a-on state. Nevertheless, exploring ultrafast TS remains a key challenge, primarily due to the limitation of real-time measurement capabilities in ps timescales. Therefore, building such a measurement capability and a systematic understanding of TS mechanism and its ultrafast transient characteristics are crucial for enabling ps programming of PCM devices.

Ever since the discovery of TS in chalcogenide semiconductor glasses^6^, several efforts have been devoted to understanding the mechanism of TS. A large number of models have been proposed to explain the TS, mainly based on thermally induced or purely electronic mechanisms^11–16^. Recently, a model explaining the electronic nature of threshold switching mechanism has also been proposed that involves the formation of metavalent bonds^17^. Though the microscopic physical mechanism of TS is still debatable, its significance is well recognized for numerous applications such as the high-speed electronic memory^2^ and selector devices^18^. They exploit the unique feature of TS as a rapid breakdown of electrical resistance at *V*_*T*_. This is observed after a finite delay time (*t*_*d*_) measured as the time between voltage exceeding the threshold value *V*_*T*_ (onset) and a steep rise in the device current (end)^6,12,19^. TS dynamics of various PCM devices show a strong voltage dependence on *t*_*d*_^16,18,20,21^. By applying electrical pulse having amplitude higher than *V*_*T*_, the *t*_*d*_ has been reduced significantly lower values to few ns^8,22–24^.

Voltage-dependent TS dynamics of Ge_2_Sb_2_Te_5_ (GST) device investigated previously demonstrate a reduction in *t*_*d*_ down to ~ 10 ns^16,21^. Even faster *set* speed of ~ 500 ps is also achieved in GST devices by using a constant low voltage incubation^9^. However, since a long (~ 10 ns) preprogramming treatment is needed before every *set* operation, the real overall *set* speed remains of the order of 10 ns. Therefore, the major challenge lies in achieving electrical switching in GST devices below 10 ns, which forms the primary goal of this paper. Threshold switching has been studied in both melt-quenched and as-deposited amorphous phases^18,19^. However, melt-quenching process is known to leave some subcritical crystalline domains in the amorphous matrix, so that the slow nucleation step is bypassed during the switching process^25^. Therefore, the crystallization of a melt-quenched GST is expected to be significantly faster than that of an as-deposited amorphous film. Along these lines, it has also been shown experimentally that the crystallization times of melt-quenched samples are an order of magnitude smaller than the as-deposited samples^26^. Hence, in order to understand the true origin of TS mechanism at the ultrafast time scales, the as-deposited samples are more appropriate choice than the melt-quenched samples. This motivated us to explore the TS dynamics of as-deposited GST devices down to ps timescale by utilizing an advanced programmable electrical test (PET) setup^19,27,28^. The PET setup has the capabilities of generating fast pulses with rise (*t*_*r*_) and fall (*t*_*f*_) times of 1 ns, and a pulse-width (*p.w.*) as short as 1.5 ns. The high-frequency contact-boards used in the PET setup are carefully designed to capture ultrafast time-resolved transient characteristics with a time resolution of 50 ps.

## Results and discussion

**Figure 1:**
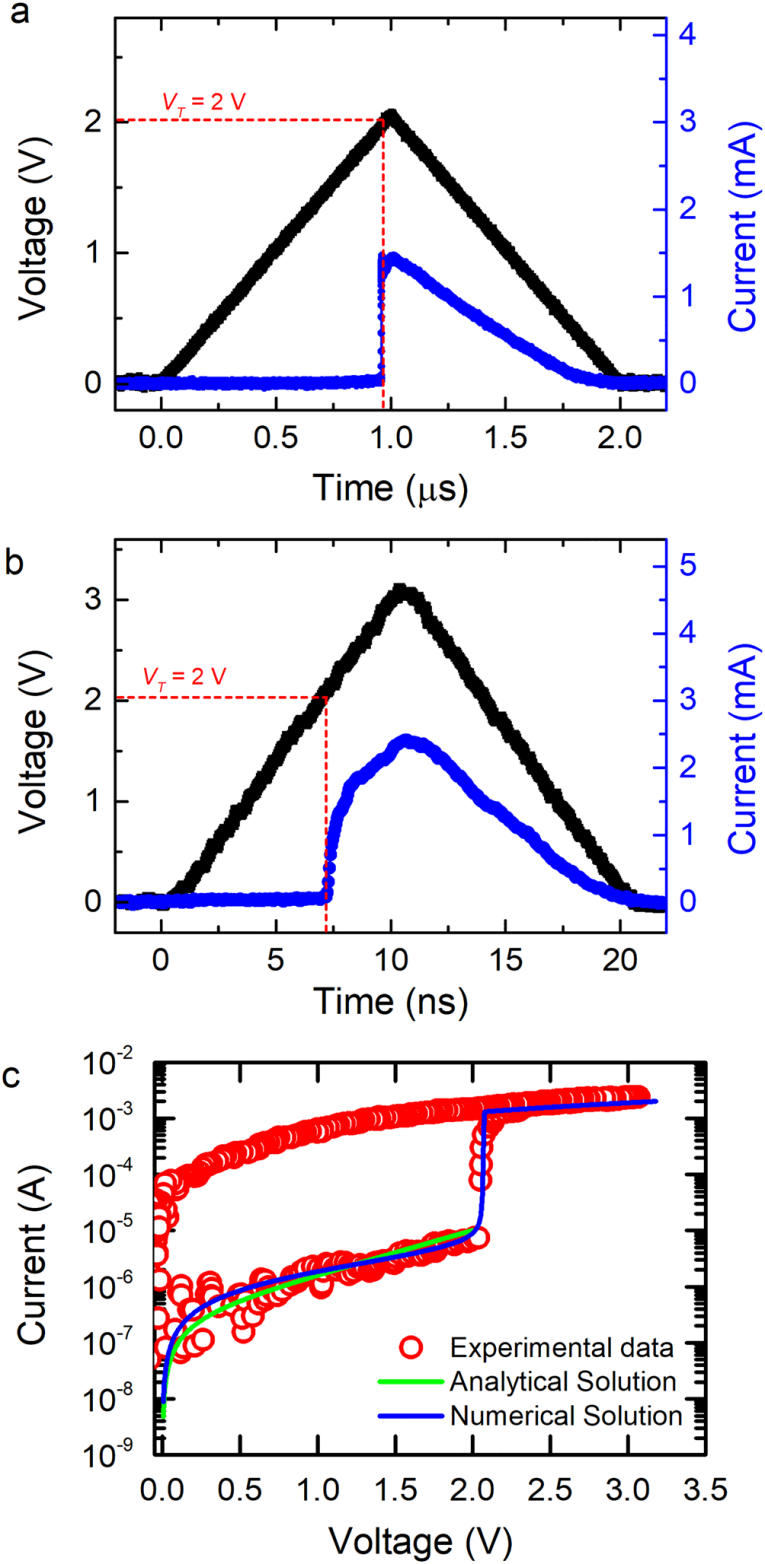
Threshold voltage of the device and its stability over the variations in applied voltage pulse parameters. (**a**) Threshold switching in GST device for a triangular pulse of 2.1 V with 1 µs leading/trailing edge. (**b**) Threshold switching for a pulse having leading/trailing edge of 10 ns. *V*_*T*_ is found to be very stable within 2 ± 0.1 V range. (**c**) I–V characteristics of GST device show the features of amorphous off state in logarithmic current scale. The conductivity rapidly increases above *V*_*T*_ in the on state, leading to set transition. The obtained experimental data was found to be in-agreement with analytical solutions^29^ in the sub-threshold conduction and also the numerical simulation based threshold-switching model^30^.

At first, it is essential to find the *V*_*T*_ (steady-state) of the GST device and examine its stability over various leading/trailing edges prior to the estimation of voltage-dependent TS dynamics and transient parameters. The steady-state *V*_*T*_ of the device is identified by the application of voltage pulse by gradually increasing the amplitude and observing the voltage at which the breakdown of electrical resistance takes place. A triangular-shaped voltage pulse of 2.1 V with long leading/trailing edge of 1 µs is applied to the as-deposited GST device as shown in Fig. 1a. Initially, the device is in high resistance a-off state as marked by very low *I*_*d*_. Upon increasing the amplitude, *I*_*d*_ remains low up to a critical voltage *V*_*T*_, above which *I*_*d*_ increases rapidly revealing a TS from a-off to on-state. This critical voltage *V*_*T*_, is found to be 2 ± 0.1 V and the corresponding threshold electric field, *E*_*T*_ of 38 ± 2 V/µm is comparable with the values reported in the literature^2,14,29^. Further, to confirm the stability of *V*_*T*_, voltage pulses of higher amplitude with faster leading and trailing edges were applied to the device (Fig. 1b). In all the cases, the device switches at *V*_*T*_ of 2 ± 0.1 V confirming the stable steady-state *V*_*T*_ (corresponding steady-state *E*_*T*_) of GST device.

Furthermore, we have employed analytical model of Ielmini and Zhang^29^ as well as that of Buscemi et al.^30^ in our study by fitting our I–V characteristics curve using the Eqs. (1) and (2) respectively.

1 $$ I = 2qAN_{t} \left( {\frac{\Delta z}{{\tau_{0} }}} \right)e^{{ - \frac{{E_{C} - E_{F} }}{kT}}} \sinh \left( {\frac{{qV_{a} }}{kT}\frac{\Delta z}{{2u_{a} }}} \right) $$

where I is device current, q is carrier charge, A is contact area, N_t_ is total trap density, ∆z is the inter-trap distance, τ_0_ is escape time, E_C_–E_F_ is the activation energy, k is the Boltzmann constant, T is the temperature, V_a_ is the applied voltage and u_a_ is the thickness of amorphous region.

2 $$ \begin{aligned} & JF = n\frac{{k\left( {T_{e} - T_{0} } \right)}}{{\tau_{R} }}, \\ & \frac{{T_{e} }}{{T_{0} }} = 1 + \frac{{\frac{{\mu q\tau_{R} }}{{kT_{0} }}F^{2} }}{{1 + \left( {\frac{{g_{T} }}{{g_{B} }}} \right)e^{{\frac{\Delta E}{{kT_{e} }} }} }} \\ \end{aligned} $$

where J is the current density, F is the electric field, n is the total carrier concentration, k is the Boltzmann constant, T_e_ is the electron temperature, μ is the mobility of the band carriers, τ_R_ is carrier relaxation time, g_T_/g_B_ is the ratio of the density of trap to band states, ΔE is the activation energy and T_0_ is the temperature.

Both these models are purely electronic in nature, employing Poole–Frenkel conduction and the resulting electric field of electrons in trap states. While these models were able to simulate sub-threshold conduction and threshold switching mechanisms, here we provide experimental validation of these models for the first time. Both the models indeed fit our experimental I–V characteristics quite well (Fig. 1c) and they give the same value of activation energy parameter of 350 meV (or the difference between the conduction band minimum and the fermi level), reiterating an electronic origin for TS mechanism at sub-ps timescales. Fitting parameters used for Eqs. (1) and (2) are provided in Supplementary Information Tables S1 and S2 respectively.

**Figure 2:**
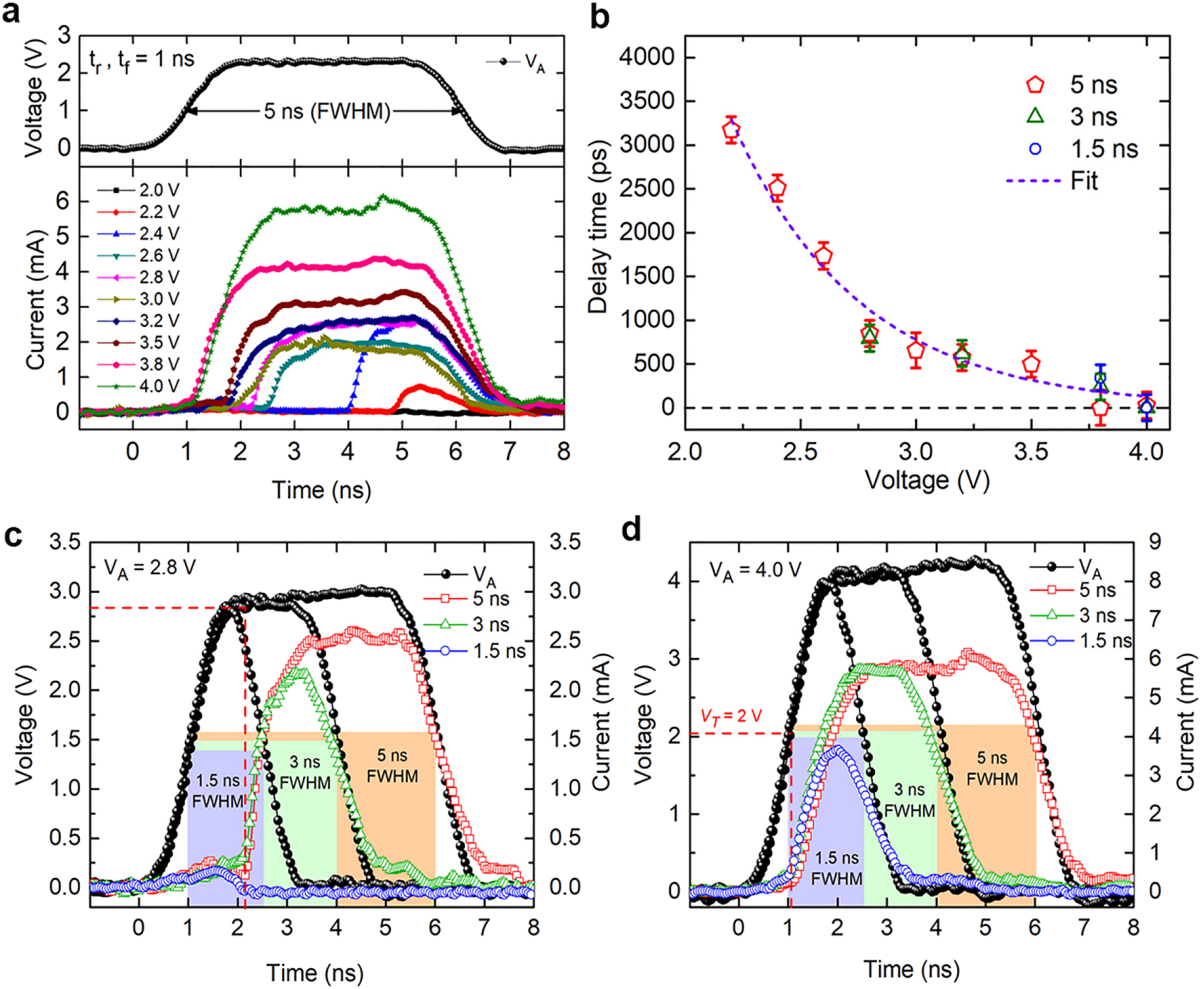
Voltage-dependent delay time characteristics for short (< 5 ns) voltage pulses. (**a**) Time-resolved measurement of *I*_*d*_ for voltage pulses with varying amplitudes (2.0–4.0 V) and a constant pulse pattern of 1 ns rise, fall time and 5 ns pulse-width. (**b**) Voltage-dependent delay time characteristics for various voltage pulses having pulse-width of 5 ns (red pentagon), 3 ns (green triangle) and 1.5 ns (blue circle). (**c**) Black spheres form voltage pulses of 2.8 V (or 4.0 V in **d**) with rectangular boxes indicating their pulse-width with red (5 ns), green (3 ns) and blue (1.5 ns) color respectively (Heights of boxes are kept different for better visibility only). Device currents corresponding to these voltage pulses show that amplitude of 2.8 V is sufficient to switch the device with pulse-width of 5 ns and 3 ns but not with 1.5 ns. (**d**) Switching event moves from plateau region to leading edge of applied voltage pulse with sufficient over-voltage. *I*_*d*_ for all three pulses show that with amplitude of 4.0 V, the device switches almost instantaneously after experiencing *V*_*T*_ without any measurable delay.

After determining the *V*_*T*_, a systematic study of TS dynamics in timescales below 5 ns is carried out. The time-resolved measurement of *I*_*d*_, for a systematic increase in the amplitude of applied voltage (*V*_*A*_) reveals an excellent TS and transient characteristics of the GST device (Fig. 2a). For *V*_*A*_ of 2.2 V and higher, the device switches within the pulse width 5 ns of the applied voltage pulse as witnessed by an abrupt increase in *I*_*d*_. With higher amplitude pulses, switching occurs promptly in the plateau region and leads to a higher on-state current. More interestingly, for sufficient over-voltages starting from 3.0 V up to 4.0 V, switching occurs within the leading edge of the pulse i.e. before the applied voltage pulse reaches to its plateau region. In order to understand the ultrafast transient characteristics of the GST device, various voltage pulses having pulse widths of 5 ns, 3 ns and 1.5 ns with systematically increasing amplitudes up to 4 V are used. Figure 2b displays the dependence of *t*_*d*_ (measured as shown in Supplementary Information Fig. S1) upon *V*_*A*_ revealing an exponential decrease of *t*_*d*_ for applied over-voltage and strikingly culminating at the lowest value of sub-50 ps as a function of increasing applied voltage. The over-voltage dependence of *t*_*d*_ can be fitted with the following equation:

3 $$ t_{d} = c_{1} \times \exp \left\{ { - \left( {\frac{{V_{A} - V_{T} }}{{V_{T} }}} \right) \times \left( {\frac{{c_{2} }}{{V_{T} }}} \right)} \right\} $$

where constant c_1_ corresponds to the *t*_*d*_ at the steady-state *V*_*T*_ and constant c_2_ indicates the order of decay of *t*_*d*_^31^. Our experimental data best fits Eq. (3) for c_1 _≈ 3944 ps and c_2 _≈ 7.9 V. Previous reports on the exponential reduction in *t*_*d*_ of GST devices upon increasing *V*_*A*_ is limited up to ~ 10 ns^6,20,32^. Whereas, the present work demonstrates the behavior of *t*_*d*_ in the ultrafast timescales that has not been previously explored and further sheds light on possibly the lowest *t*_*d*_, which will enable strikingly fast TS thereby improving the speed of *set* process in PCM devices. The delay time measured or the set pulse used in previously reported studies on GST and various other phase change materials is compared in Table 1, where present work outperforms with more than two orders faster TS in GST devices.

**Table 1:** Comparison of present work with existing literature.

Material	Delay time (min.)	Set pulse duration (min.)	References
Ge_1_Sb_2_Te_4_	100 ns	–	^20^
Ge_2_Sb_2_Te_5_	10 ns	–	^21^
Ge_2_Sb_2_Te_5_ (doped)	8 ns	–	^16^
GeTe_6_	5 ns	–	^18^
Nitrogen-doped Ge_2_Sb_2_Te_5_	–	3 ns	^23^
GeTe	–	1 ns	^22^
Sc_0.2_Sb_2_Te_3_	–	0.7 ns	^8^
Ge_2_Sb_2_Te_5_	–	0.5 ns (with priming)	^9^
Ge_2_Sb_2_Te_5_	50 ps	1.5 ns	This work

In order to elucidate the nature of ultrafast transient characteristics and occurrence TS during the leading edge of V_A_, we have chosen two different amplitudes such as *V*_*A*_ of 2.8 V and 4.0 V. It can be clearly observed from Fig. 2b that the *t*_*d*_ for V_A_ of 2.8 V is 800 ps and that for 4.0 V, it is less than 50 ps. Figure 2c displays TS characteristics for *V*_*A*_ of 2.8 V (black dots) with different pulse-widths such 5 ns, 3 ns and 1.5 ns. It is important to note that the device exhibits TS for pulse widths of 3 ns (corresponding *I*_*d*_, marked in green dots) and 5 ns (corresponding *I*_*d*_, marked in red dots) as evidenced by a rapid increase of device currents. On the other hand, for 1.5 ns pulse width, the device does not exhibit TS (corresponding *I*_*d*_, marked in blue dots). This is because for V_A_ of 2.8 V the *t*_*d*_ is 800 ps, which is insufficient to meet the required time for TS above the actual *V*_*T*_ of 2.0 V till the plateau region. Furthermore, Fig. 2d depicts TS characteristics for the *V*_*A*_ of 4.0 V (black dots) having different pulse-widths such 5 ns, 3 ns and 1.5 ns. It is interesting to note that these devices exhibit TS for various pulses having all of the pulse widths such as 5 ns (corresponding *I*_*d*_, marked in red), 3 ns (corresponding *I*_*d*_, marked in green) and 1.5 ns (corresponding *I*_*d*_, marked in blue), as evidenced by a rapid increase of device currents at *V*_*T*_ of 2.0 V itself. It is noteworthy to mention here that the GST device exhibits TS at a remarkable speed at *V*_*T*_ without any measurable delay (less than 50 ps) for the *V*_*A*_ of 4.0 V, which is equal to twice of *V*_*T*_ (see Supplementary Information, Fig. S2).

**Figure 3:**
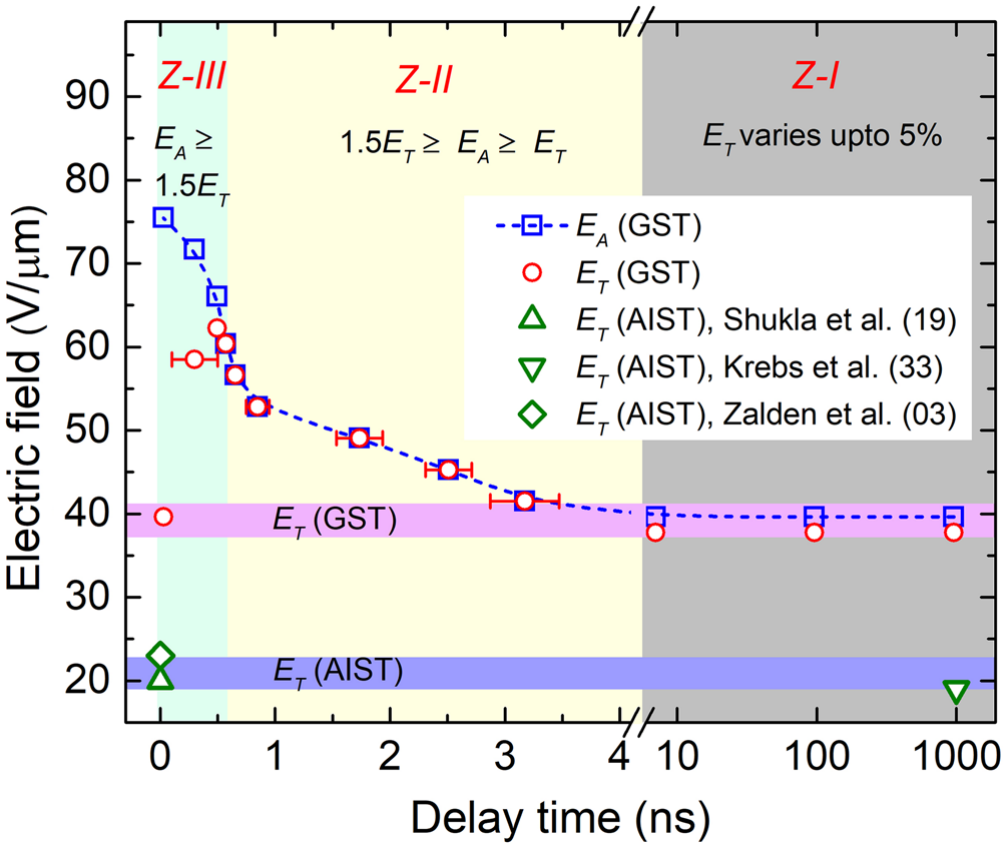
Trajectory map of electric field required for ultrafast switching. Threshold electric field is categorized in three zones for GST: Zone-I is for TS with longer *t*_*d*_ of more than 10 ns, for the *E*_*A*_ almost equal to *E*_*T*_. Zone-II is for *E*_*A*_ between *E*_*T*_ and 1.5 times of *E*_*T*_ where the device switches within the plateau region of the pulse, and data points of *E*_*A*_ and *E*_*T*_ coincides. Zone-III is for *E*_*A*_ more than 1.5 times *E*_*T*_, where device switches within the leading edge of the pulse. This behavior of threshold switching in GST device is quite different than that of AgInSbTe devices where *E*_*A*_ required for ultrafast switching is same as its steady-state *E*_*T*_.

Based on the above experimental results, a trajectory map (Fig. 3) has been constructed with the following three possibilities of switching dynamics of GST device; (1) the device exhibits TS with longer *t*_*d*_ of more than 10 ns, for the *E*_*A*_, which is almost equal to *E*_*T*_, (2) the device exhibits TS with the *t*_*d*_ below 4 ns down to 600 ps, for the *E*_*A*_ in the range from *E*_*T*_ up to 1.5 *E*_*T*_, (3) the device exhibits TS with the *t*_*d*_ below 600 ps until sub-50 ps, for the *E*_*A*_ in the range from 1.5*E*_*T*_ up to 2*E*_*T*_. Furthermore, this map enables various possibilities of the speed of TS based on the *E*_*A*_ as marked by three different zones. More interestingly, the dependence of TS on *E*_*A*_ of GST device is different from that of AgInSbTe (AIST) device, where the *E*_*A*_ required for TS is the same as that of *E*_*T*_^3,19,33^. Hence, these two different types of TS dynamics of GST and AIST phase change materials are presented in Fig. 3, which will be useful for making the appropriate choice of material suitable for the energy efficient TS with its relevant speed.

The above experimental results pose an important question: what governs the onset of TS in GST at such ultrafast timescales? The origin of TS in amorphous chalcogenides is still debated and divided into two schools of thought, namely the thermal and electronic models^11–14,34^. In the thermal model, field-induced nucleation of the needle-shaped conductive crystalline channel is the key to switching from a high- (OFF) to a low-resistive (ON) state. The growth of this conductive channel is activated by an energy barrier for nucleation (W) and a characteristic nucleation voltage (*Ṽ*) (different from threshold voltage *V*_*T*_, *Ṽ* <  < *V*_*T*_). Below *Ṽ*, the nucleation switching fails, leading to non-conducting spherical particles. Though this model can successfully describe the switching behavior close to *Ṽ*, it may not be applicable to the present scenario for the following reasons: 1. This model is focused on under-threshold switching close to *Ṽ* as against the threshold or over-threshold voltages applied in the present study. Hence, the delay times predicted by this model for infinite bias voltage are several orders higher than our experimental results for *V*_*A *_≈ 2*V*_*T*_ (*t*_*d*_ < 50 ps). Moreover, it is well known that the delay times at the threshold voltage are highly statistical and no significant current flows below the threshold voltage^6^. 2. If the switching is nucleation activated, then the *I*_*d*_ for voltages above *Ṽ* must steadily increase even during the delay time as the nucleus grows in size, whereas in our case *I*_*d*_ remains constant until the onset of switching for applied voltages *V*_*A*_ ≥ *V*_*T*_ (see Supplementary Information Fig. S3). 3. In the thermal model, the switching behavior is described at 160–170 °C, well above crystallization temperature that can very well lead to the formation of crystal nuclei. In contrast, the results presented in this study are for ambient conditions. Studies that renewed interest in nucleation theory on GST based PCMs subjected to non-favorable conditions like the high nucleation barrier and temperatures low-enough than the glass transition temperature are also known. However, unlike our present case, these studies are focused on under-threshold voltages and such conditions are expected to increase the delay times to several tens of seconds^35^. Hence, in the ultrafast timescales of 1.5–5 ns pulse width of the *V*_*A*_ and *t*_*d*_ of sub-50 ps observed in this study, we believe that the nucleation of crystals is highly unlikely.

The highly disordered nature of the atomic structure in chalcogenide glasses leads to a large number of unsaturated and oversaturated chemical bonds giving rise to localized defect bands or charge traps in the electronic structure. These traps are believed to lie about 0.1–0.3 eV above (below) the highest occupied (lowest unoccupied) energy bands. The high concentration of these acceptor (donor) bands tend to pin the Fermi level near the middle of the energy gap region, control the carrier mobility, form regions of space-charge at the interface, and also act as centres of recombination^34,36,37^. Such charge traps are also well known to affect carrier mobility and recombination rates in hydrogenated amorphous silicon (a-Si:H), leading to poor solar cell efficiencies^38^. In chalcogenide glasses, the carrier transport is considered to be trap limited in the form of hopping between the trap states by tunneling or Poole-Frankel (PF) mechanism, or a combination of both^13,29,39^, or by small polarons^40^. The electronic model for switching is based on the instability in the carrier transport caused by an increasing applied electric field. At a critical electric field, the switching event in the form of tunneling or a field-ionizing carrier is initiated at one of the electrodes. As the random event is initiated, the discrete traps (acceptor and donor bands) begin to localize charge carriers. Once the carriers are trapped, a potential barrier is created so that no more carriers can be accommodated in the trap and then the whole transport process is controlled by drift–diffusion and Shockley–Read–Hall (SRH) e–h recombination process. A conductive state is reached when the rate of generation exceeds the recombination and then net current flows through the material. The carriers are then collected at the other electrode after a certain *t*_*d*_. Hence, the *t*_*d*_ could be interpreted as the time required for the propagation of carriers from one electrode to the other mediated through the trap states, after a random event has occurred. This *t*_*d*_ is highly statistical near the *V*_*T*_ and is significantly lower at higher *V*_*A*_.

**Figure 4:**
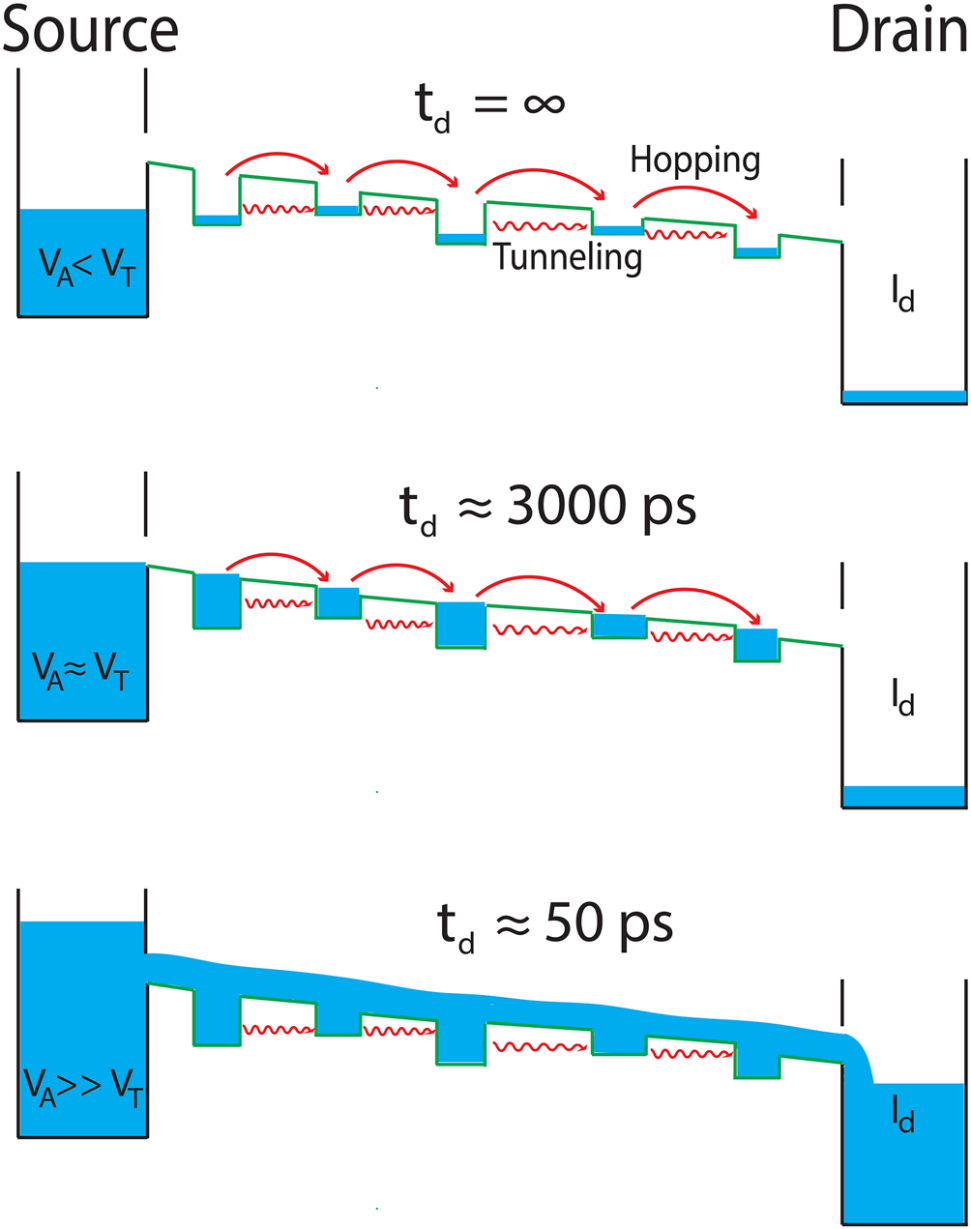
Water filling model for threshold switching in ultra-fast timescale. As *V*_*A*_ < *V*_*T*_, carrier transport is limited by a potential barrier caused due to the trap states, and device current is negligible. With *V*_*A*_ = *V*_*T*_, switching event initiates at one of the electrodes and carriers gain sufficient mobility to travel from one electrode to the other leading to a large current. Delay time can be considered as the time required by charge carriers to travel from one electrode to the other. When the applied voltage is higher than the threshold voltage, more carriers can reach the opposite electrode leading to larger current.

In the present study, using our experimental results we have shown that (a) the *t*_*d*_ exponentially drops as a function of *V*_*A*_ above *V*_*T*_, (b) the steady-state current increases as the function of the over-voltages and (c) higher voltages are required to see the switching mechanism when the pulse width is extremely narrow (1.5 ns). These behaviors can be understood from a simplistic water filling model, a schematic of which is shown in Fig. 4. Let us assume that the water flows from source to drain through a series of traps. Classically, no water is collected at the drain when the level of water is below a certain threshold. Above the threshold level, water begins to flow, fills traps, and finally gets collected at the drain after a delay time, the time required to fill the traps. Over-threshold levels in the source can result in water coming out with greater pressure leading to filling of traps sooner. Similarly, when a voltage pulse is applied, *t*_*d*_ will depend on the applied voltage. Higher the *V*_*A*_, the faster will be the rate of filling of traps, leading to smaller delay times. But in the case of electrons, additional effects are also possible. The electrons can tunnel through the barrier even though their energy is lesser than the barrier height, and hence there is always a very small current even for *V*_*A*_ < *V*_*T*_. Moreover, the PF effect can lead to band bending as a function of the electronic field, from source to drain. This can result in the reduction of the potential barrier on one side of the trap, the barrier towards the higher electric field will be higher (Fig. 4). As the trap states are only few hundreds of meV deep, this band bending can further help in thermally assisted electron transport over the potential barrier, which can also contribute to a small device current. Thus for all voltages below the actual *V*_*T*_, we see a small drain current. At *V*_*T*_, the *V*_*A*_ is just sufficient to fill the carrier traps and balance out the generation-recombination processes. So, the carriers have very minimal mobility to reach the other electrode. However, over-voltages can help the carriers gain higher mobility resulting in the rapid filling of the traps. When the *V*_*A*_ is higher than the threshold voltage, more carriers can reach the opposite electrode leading to larger current. At extremely narrow pulse widths (≤ 1.5 ns) if the voltage is not sufficiently high (2.8 V, Fig. 2c), the carriers are unable to fill the traps and reach the opposite electrode within the duration of pulse, due to limited mobility. So, in order to realize switching in this case, higher carrier mobility is required, which can be achieved by providing excess over-voltage (4.0 V, Fig. 2d).

Though the electronic and thermal theories have contrasting philosophies, both agree that the voltage dependence of time delay is exponential. However, for the reasons discussed above, we believe that the thermal effects have a minimal role in the onset of threshold switching, at least in the extremely fast timescales of sub-50 ps as observed in GST as well as In_3_SbTe_2_ devices^41^. We also calculated the minimum time taken (*t*_*min*_) by a charge carrier under the ballistic regime to transit through the chalcogenide film of thickness L = 53 nm and mobility µ = 1 cm^2^/(V s) using the relation *t*_*min*_ = L/(E × µ) where E is the electric field. For *E*_*A*_ = 75.5 V/µm, one would expect *t*_*min*_ = 7 ps, which is comparable to the observed delay time of less than 50 ps for twice the *V*_*T*_. It should be noted that the delay time for *V*_*A*_ = 4 V could be much smaller than the observed value since the measurement capability of PET setup is limited to 50 ps. Similar experimental evidence has been reported for AgInSbTe devices^3^, where ps TS is achieved using THz pulse demonstrating purely electronic nature of TS.

In conclusion, we have demonstrated the electric field dependent transient characteristics of GST devices in the picosecond timescale. A trajectory map is defined to provide a clear correlation for the electric field required to switch the GST devices within specific time duration. The map demonstrates that at a sufficiently large applied voltage (two times of *V*_*T*_), the device switches almost immediately after experiencing the *V*_*T*_ without any measurable *t*_*d*_ (i.e. *t*_*d*_ < 50 ps). Furthermore, the ultrafast threshold switching achieved in these devices provides strong evidence for the electronic nature of the threshold switching process, which is evidenced by constant device current during the delay time. Therefore, the picosecond threshold switching dynamics and its detailed mechanism presented here offer new insight for the design of improved PCM with enhanced capabilities.

## Methods

GST devices were fabricated using radio frequency (RF) magnetron sputtering in sandwich-type structure^19^ on SiO_2_ substrates as shown in Supplementary Information Fig. S4a. Substrates were pre-cleaned by ultrasonic agitation with acetone and propanol. A thin PCM layer of GST (using 30 W RF power at 10 sccm Ar flow and 10 rpm substrate rotation speed with a deposition rate of 0.0627 nm s^−1^) is deposited between two metal contacts of Titanium (using 60 W RF power at 10 sccm Ar flow and 10 rpm substrate rotation speed with a deposition rate of 0.0086 nm s^−1^). Mechanical masks were used to make specific patterns of contacts and the active layer. A similar trend of voltage-dependent threshold switching dynamics has also been observed on GST devices fabricated using optical lithography technique as shown in Supplementary Information Fig. S4b. The amorphous nature of as-deposited thin films was confirmed using X-ray diffraction and thickness was measured as 53 nm using X-ray reflectometry as shown in Supplementary Information Fig. S5.

For time-resolved measurement of threshold switching dynamics of GST device, an advanced programmable electrical test (PET) system^19,27,28^ is employed that essentially consists of Arbitrary Waveform Generator (AWG), Digital Storage Oscilloscope (DSO) and a custom-made probe station with high-frequency contact-boards having Impedance Matching Circuit (IMC) and an amplifier (Amp) circuit. AWG (*Agilent Technologies—81160A*) has the capability of generating voltage pulses as fast as having 1 ns rise, fall time and as short as 1.5 ns FWHM (Full Width Half Maximum) pulse-width. DSO (*Teledyne Lecroy—Wavepro735zi-A*) has the specifications of measuring the signal with 3.5 GHz bandwidth and it can capture the device response in fast timescale with 50 ps resolution. In order to perform switching from as-deposited amorphous state, each time only one pulse is applied on a cell, and then it is discarded.

## Supplementary Information

Supplementary Information
