# Supplementary material for: A scheme for enabling the ultimate speed of threshold switching in phase change memory devices
Source: Sci Rep. 2021 Mar 17;11:6111. doi: 10.1038/s41598-021-85690-9 (PMC7969762; doi:10.1038/s41598-021-85690-9)
Supplement: Supplementary file 1 — Supplementary Information [file 41598_2021_85690_MOESM1_ESM.pdf]

Supplementary Materials for

A scheme for enabling the ultimate speed of threshold switching in phase change memory

Nishant Saxena,[1] Rajamani Raghunathan,[2] and Anbarasu Manivannan[1,*]

[1]Department of Electrical Engineering, Indian Institute of Technology Madras, Chennai -600036, India.

[2]UGC-DAE Consortium for Scientific Research, DAVV Campus, Khandwa Road, Indore - 452001, Madhya Pradesh, India.

[*]Correspondence to: anbarasu@iitm.ac.in

This PDF file includes:

Supplementary Text

Supplementary Figures S1 to S5

Supplementary Tables S1 to S2

Delay time in Phase Change Memory Devices

When the applied voltage exceeds a threshold voltage (V_T), the device switches from amorphous-off (a-off) to amorphous-on (a-on) state after a certain delay time (t_d). Most of the delay time studies done so far utilize rectangular shaped voltage pulses having > 100 ns pulse-width (*p.w.*). In these cases, rise time (t_r) of the pulse is very small as compare to the *p.w.* and is insignificant for the calculation of t_d. Therefore, t_d has been considered as the time elapsed prior to initiation of the switching event. However, there are two major concerns: (i) for short pulses, t_r becomes comparable to the *p.w.* and (ii) by increasing the applied voltage, t_d can be reduced down to a value where device may switch within the leading edge of the pulse. Hence, for study of ultrafast switching dynamics at picosecond timescale, a precise measurement of t_d is required. Therefore, t_d is measured as the time interval between the events of device experiencing the V_T and steep rise in I_d. Figure S1 shows t_d for different cases (A) for $V_A = V_T$, (B) for $V_A >> V_T$.

Ultrafast threshold switching and *set* operation within 1.5 ns pulse-width

Threshold switching and associated delay time can be controlled by the applied voltage (or electric field). With sufficient over-voltage (4 V), the device switches extremely fast without any measurable delay (< 50 ps). Such ultrafast threshold switching and *set* operation have been achieved within a minimum pulse-width of 1.5 ns. Time-resolved measurement of applied voltage and device current are shown in Fig. S2. The device switches at V_T of 2 ± 0.1 V (corresponding E_T = 38 ± 2 V/µm) and device current increases abruptly owing to the breakdown of electrical resistance of the device. Upon removal of the pulse (trailing edge), device current follows the applied voltage indicating low resistance *set* state, which is confirmed by subsequent read pulse. More than two orders of magnitude change in resistance of the device before and after *set* pulse can be observed in Fig. S2.

Constant current during the delay time

The delay time can be interpreted as the time required for the propagation of charge carriers from one electrode to the other mediated through the trap states, after a random event has occurred. Our results clearly demonstrate that the device current during delay time is very small as compare to the on state current and remains almost constant during the entire duration of delay time as shown in Fig. S3 (a) for GST device and (b) for In_3SbTe_2 device.

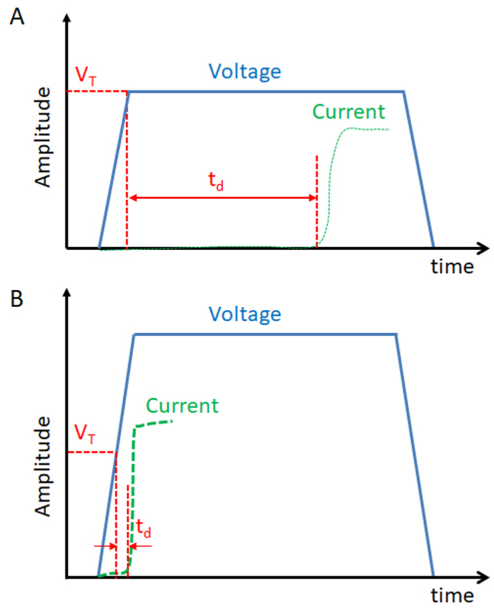

Supplementary Figure S1. Measurement of delay time. (A) $V_A = V_T$, device switches in plateau region (B) $V_A >> V_T$, device switches within leading edge (rise time).

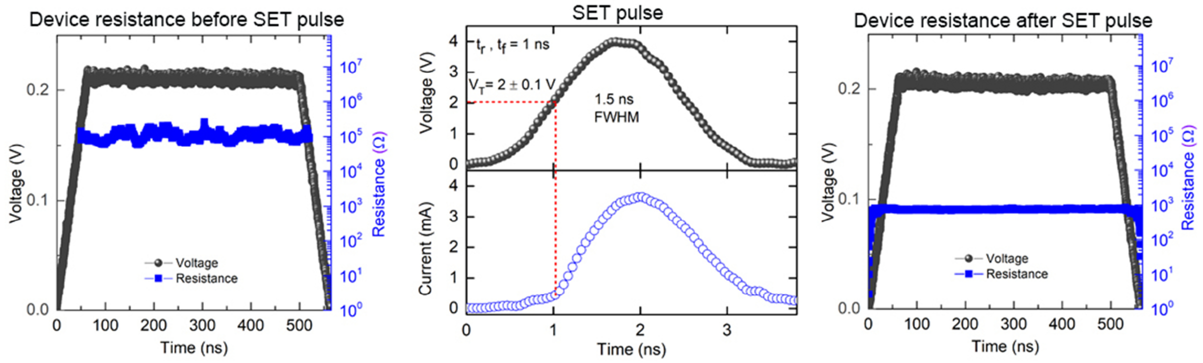

Supplementary Figure S2. Ultrafast threshold switching and set operation in 1.5 ns. Time-resolved measurement of applied voltage and device current with change in device resistance before and after the set pulse.

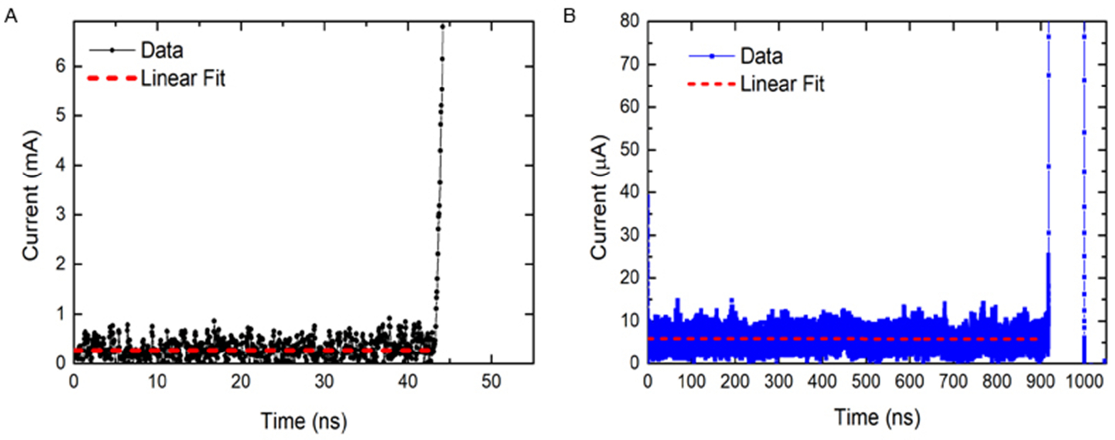

Supplementary Figure S3. Constant current observed during delay time. (A) in GST device, (B) in InSbTe device.

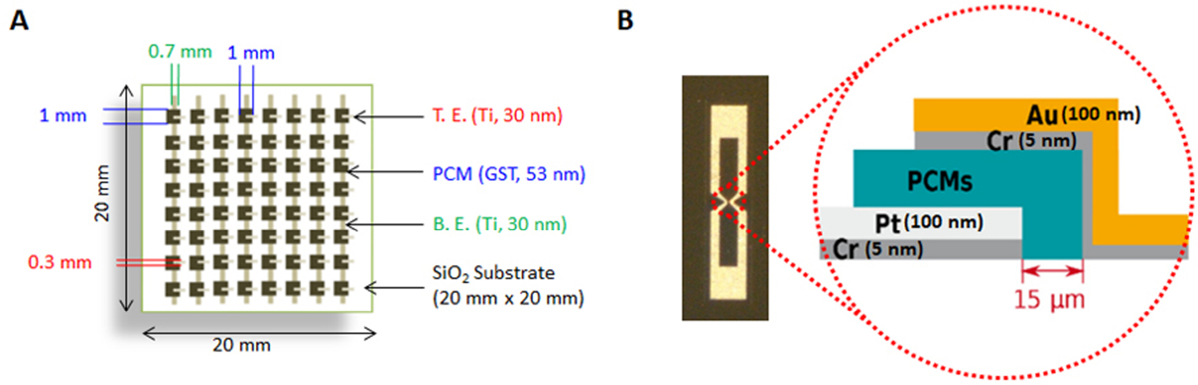

Supplementary Figure S4. Schematic of PCM device. Device fabricated using (A) mechanical masks, (B) lithography technique.

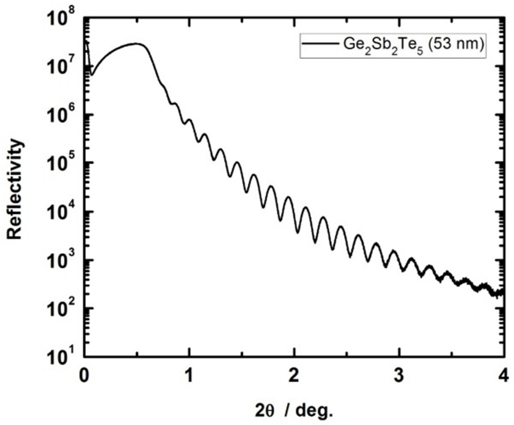

Supplementary Figure S5. X- ray reflectivity measurement of as-deposited GST thin film.

Thickness of the GST thin film calculated using x-ray reflectivity plot is found to be 53 nm.

Supplementary Table S1. Matching experimental I-V curve with subthreshold conduction model[29]: Experimental data is best fitted by equation (2) for the following parameters:

**Parameter**	**Value**
Activation energy (E_C-E_F)	0.35 eV
Intertrap distance (Δz)	5 nm
Thickness of amorphous layer (u_a)	53 nm
Total trap density (N_t)	2.5×10^{20} cm^{-3}
Escape time (t_0)	1×10^{-15} s
Boltzmann Constant (k)	8.617×10^{-5} eV/K
Temperature (T)	300 K
Carrier charge (q)	1.6×10^{-19} C

Supplementary Table S2. Matching experimental I-V curve with numerical solution based threshold switching model[30]: Experimental data is best fitted by equation (3) for the following parameters:

**Parameter**	**Value**
Activation energy (ΔE)	0.35 eV
Mobility of band carriers (μ)	2.1 $cm^2V^{-1}s^{-1}$
Carrier relaxation time (τ_R)	1×10^{-12} s
Ratio of density of trap to band states (g_T/g_B)	5×10^{-4}
Total carrier concentration (n)	1×10^{21} cm^{-3}
Amorphous chalcogenide thickness (u_a)	53 nm
Boltzmann constant (k)	8.617×10^{-5} eV/K
Temperature (T_0)	300 K
Electronic charge (q)	1.6×10^{-19} C
